# Edema in childhood nephrotic syndrome: possible genes–hormones interplay

**DOI:** 10.1186/s43141-022-00310-x

**Published:** 2022-02-18

**Authors:** Hanan El-Halaby, Ashraf Bakr, Riham Eid, Hussein Abdelaziz Abdalla, Nashwa Hamdy, Nora Shamekh, Amira Adel, Ahmed El-Husseiny

**Affiliations:** 1 Pediatric Intensive Care Unit, Pediatrics Department, Mansoura University Children’s Hospital, Faculty of Medicine, Mansoura University, Mansoura, Egypt; 2 Pediatric Nephrology Unit, Pediatrics Department, Mansoura University Children’s Hospital, Faculty of Medicine, Mansoura University, Mansoura, Egypt; 3 Medical Biochemistry Department, Faculty of Medicine, Mansoura University, Mansoura, Egypt; 4 Pediatrics Department, Mansoura University Children’s Hospital, Faculty of Medicine, Mansoura University, Mansoura, Egypt

**Keywords:** Children, Nephrotic syndrome, Atrial natriuretic peptide, Gene polymorphisms

## Abstract

**Background:**

The role of atrial natriuretic peptide (ANP) in edema formation in idiopathic nephrotic syndrome (INS) was studied before with conflicting results reported; however, the possible contribution of genes regulating ANP expression and receptors was never explored.

**Methods:**

One hundred children (60 with active INS and 40 in remission) were studied for plasma atrial natriuretic peptide (ANP), urinary sodium, ANP gene A2843G and ScaI polymorphisms, and natriuretic peptide receptor clearance C (-55) A polymorphism. For comparative purposes, 20 healthy controls were studied for ANP levels.

**Results:**

ANP was higher in active compared to remission patients (*p*<0.001). ANP in the healthy control group was significantly lower than the ANP level of active INS (during edema) group (*p*=0.009) but did not show significant differences when compared to ANP levels of either active INS group after resolution of edema or remission group (*p*= 0.42 and 0.56, respectively). Urinary sodium levels in edematous patients were significantly lower while ANP levels were significantly higher during edema than after resolution (*p*< 0.001 for both). Genotypes’ frequencies of studied polymorphisms did not differ between active and remission groups. Patients with the A1A1 genotype of ScaI polymorphism had higher ANP levels compared to other genotypes (*p* =0.01).

**Conclusions:**

During edema, ANP levels are elevated in INS children however this increment is not associated with natriuresis suggesting a blunted renal response to ANP. Polymorphisms of genes regulating ANP levels and receptors don’t seem to be implicated in edema formation except for the A1A1 genotype of ScaI polymorphism however, its possible role needs further evaluation.

## Background

Idiopathic Nephrotic syndrome (INS) is the most common glomerular disorder in childhood [1] with edema representing its typical clinical presentation. There are 2 suppositions to clarify edema pathophysiology in INS; the “underfill” hypothesis which relies on the decrease in oncotic pressure resulting in excess shifting of fluid out of the intravascular to the interstitial space, causing hypovolemia, decreased kidneys perfusion, stimulation of the renin–angiotensin–aldosterone system, ending in sodium retention [2]. Alternatively, the “overfill” hypothesis relies on the primary renal sodium retention [3]. Thus, the complete explanation of edema pathogenesis and its persistence is not fully declared and is a complex interplay of various factors.

The contributory role of plasma atrial natriuretic peptide (ANP) concentration, as a volume regulatory hormone, in edema formation in INS was widely studied before [4–6]. ANP acts on blood vessels and kidneys inducing natriuresis and diuresis in distal convoluted tubules and collecting ducts [7]. However, conflicting results of ANP levels in different children with INS were reported [6, 8, 9]. Some investigators have demonstrated a blunted responsiveness to ANP despite higher-than-normal circulating plasma levels of ANP which might be caused by overactive efferent sympathetic nervous activity, as well as an enhanced tubular breakdown of cyclic guanosine monophosphate [10].

Polymorphisms of genes controlling ANP and its receptor expression as ANP A2843G and A188G, ScaI polymorphism of ANP gene, and the natriuretic peptide clearance receptor (NPRC) gene polymorphism C (-55) A were studied in some disorders as essential hypertension and chronic heart failure [11, 12] but never been explored before in INS.

This study was carried out to further explore the role of ANP in INS edema formation and renal ANP resistance hypothesis. Additionally, for the first time, the possible role of three polymorphisms controlling ANP and its receptors expressions in pathogenesis and outcome of edema in childhood INS was explored.

## Methods

### Patients

This pilot study included children with INS admitted to the pediatric nephrology unit at Mansoura University Children’s Hospital over a period of 3 years (July 2015 to June 2018). Ninety-three children with INS (in relapse) were admitted during this period; of them, 60 were enrolled in the study as an active INS group. Forty patients in remission were recruited from outpatient clinics as the second group (remission group). A group of 20 age and sex-matched healthy children (no history or clinical findings suggestive of kidney or cardiac disorders, normal blood pressure, and urine analysis screening) were included to establish a normal reference range for serum ANP for comparative purposes. Exclusion criteria included (33 patients): children with congenital (*N*=6) or infantile NS (*N*=5), secondary NS (*N*=3), patients with impaired kidney function (*N*=9) or active infection (*N*=6) at the time of admission, and those who refused to participate in the study(N=4). Nephrotic syndrome was defined as per the “International Study for Kidney Diseases in Children” criteria [13]. Active NS, defined as the presence of nephrotic range proteinuria [urine protein/creatinine (UPC) ratio > 2]. Steroid resistant nephrotic syndrome (SRNS), Steroid responsive NS included: Steroid-dependent nephrotic syndrome (SDNS), Frequent relapsing nephrotic syndrome (FRNS), and infrequent relapsing NS (IFRNS) were defined as per “Kidney Disease Improving Global Outcomes” Guidelines [14]. The patient’s body weight, blood pressure, pulse rate, and degree of edema were assessed on admission day. Hypertension was defined as an average systolic blood pressure (SBP) and/or diastolic blood pressure (DBP) that is greater than or equal to the 95th percentile for sex, age, and height on three or more occasions. Blood samples were drawn to measure serum albumin, creatinine, and cholesterol. Urine samples were collected for urinary protein, creatinine, and sodium. Glomerular filtration rate was calculated using the Schwartz formula.

### Plasma Atrial Natriuretic Peptide (ANP) concentration

Plasma ANP concentration was measured by the use of enzyme-linked immunosorbent assay (ELISA) technique via sandwich immunometric assays kits (Sunred Biological Technology Co., Shanghai) for in vitro quantitative measurement of ANP concentrations in plasma. This assessment was done once for children in remission. For the active INS group, ANP levels were determined once on admission and then after the resolution of edema.

### Single nucleotide polymorphism genotyping

**Fig. 1:**
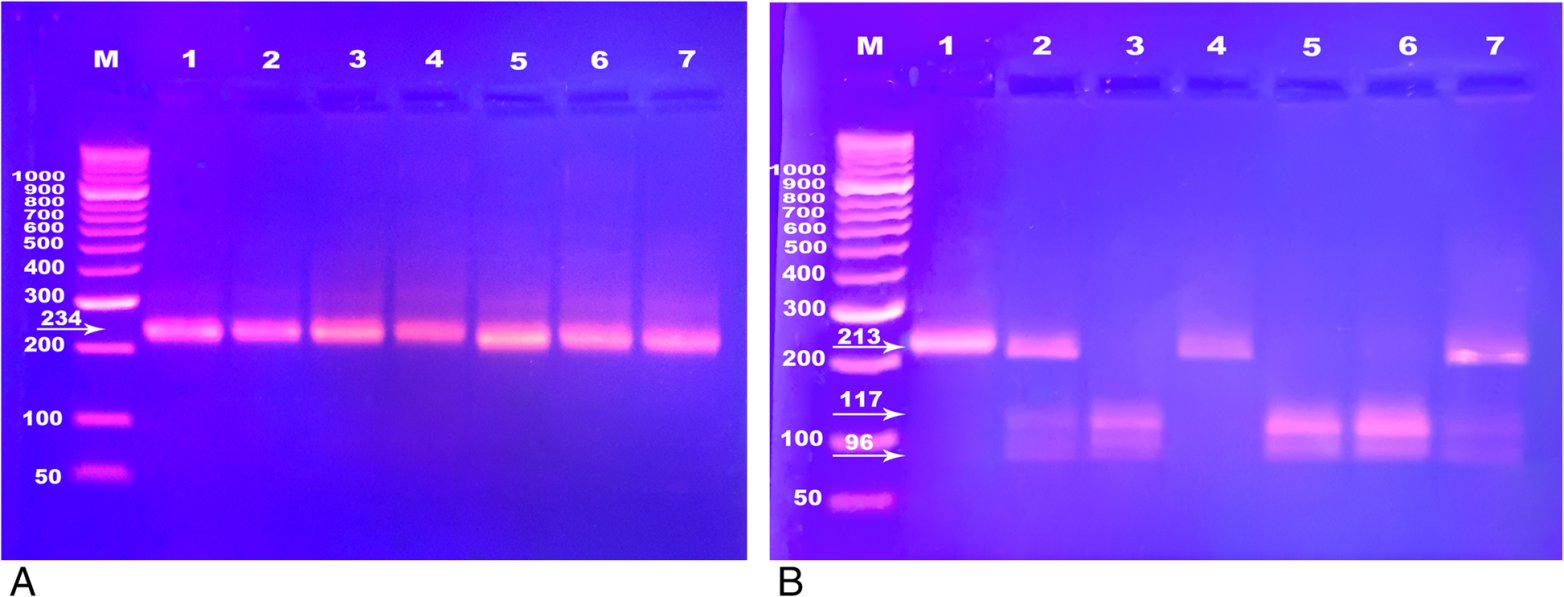
PCR-RFLP genotyping of ANP ScaI polymorphism. **A** Showing visualization of 234 bp PCR product by 2% agarose gel electrophoresis, Lane M represents DNA ladder, lanes 1-7 PCR products represented by one band at 234 bp. **B** Showing RFLP analysis of ANP ScaI polymorphism by 3% agarose gel electrophoresis; A1A1 genotype produces 2 fragments 213 and 21 bp, A2A2 genotype produces 3 fragments 117, 96, and 21 bp (lanes 3, 5, and 6), while A1A2 produces 4 fragments 213, 117, 96, and 21 bp (lanes 2 and 7), realize that 21 bp fragment couldn’t be visualized as it’s too light and run out the gel, lane M represents DNA ladder

All participants were screened for the ANP gene (A2843G) polymorphism as described by Xue et al. [12], the NPRC C (-55) A polymorphism as described by Sarzani et al. [15], and ScaI polymorphism of ANP gene as described by Gruchala et al. [16]. The genomic DNA of blood samples was extracted by EZ-10 Spin Column kits, Markham, Canada according to manufacturer instructions. The polymorphisms were genotyped by PCR-based restriction fragment length polymorphism (RFLP). PCR mixture of 12.5 μl of the PCR master mix (2x) (Thermo-Scientific Dream Taq Green PCR Master Mix, Thermo Fisher Scientific Inc, Lithuania, EU), 5 μl of the template DNA, 1.5 μl of the forward primer, 1.5 μl of the reverse primer, and 4.5 μl of the nuclease-free water to reach a total volume of 25 μl. Thermal cycler (Techne TC-312, UK) was programmed according to each polymorphism amplification program. After amplification, agarose gel was used to separate the PCR products. The PCR product of each gene was digested by the corresponding restriction enzyme and agarose gel was used to separate the products of digestion and detect the different genotypes. The primers and restriction enzymes used are presented in Table 1. Figure 1 shows PCR-RFLP genotyping of ANP ScaI polymorphism.

**Table 1:** The primers and restriction enzymes used for PCR-RFLP genotyping

SNP	Primer sequence	Size of PCR product	Enzyme	Fragments
**ANP gene (A2843G)**	**Forward** 5′-TAGGGATGA-TCGTTGCTGACTTTG-3′ **Reverse** 5′-GAGTGACCTT-TTGCCTTGGATT-3′	125 bp	Hinf1	103 and 22 bp
**NPRC C (-55)A**	**Forward** 5′-CACCGTCAATTACAAACACTTGGACAAGTCTAAC-3 **Reverse** 5′-CACCCTTCCTCTTTCCTCCCCACTCTTCTCTCCA-3	370 bp	HgaI	177, 105, and 88 bp or 265 and 105 bp
**ANP gene (ScaI)**	**Forward** 5′-GGT GGGAAGCAGGTGGTCAGTACTCAAGTTCAG AGGATG GGC-3′′ **Reverse.** 5′-CAC AAC TCC ATG GCA ACAAGA TGA CAC AAA TGC-3′.	234 bp	ScaI	117, 96, and 21 bp or 213 and 21 bp

*SNP* single nucleotide polymorphism, *ANP* atrial natriuretic peptide, *NPRC* natriuretic peptide receptor clearance, *PCR-RFLP* polymerase chain reaction-restriction fragment length polymorphism

### Statistical analysis

The patients’ data are presented as numbers (percentage), means, and standard deviation. Differences in genotype and allele frequencies were evaluated by chi-square or Fisher’s exact tests. The patients and controls were compared using Student’s *t*, chi-square, and Fisher’s exact tests. ANP and urinary sodium levels before and after edema resolution were compared using paired *t*-test. A *p*-value of less than 0.05 was considered significant. The statistical analysis was done by IBM® SPSS® Statistics SPSS (Statistical Package for Social Sciences) version 25.

## Results

The clinical and laboratory data of all 100 children with NS included in the study are presented in Table 2. Serum ANP before and after treatment of edema in the active NS group are presented and compared to those of the NS in remission group. A group of 20 healthy children (13 males and 7 females) aged 6.2±1.9 years were evaluated for serum ANP which was 96.5±21.7 (ng/ml) and was significantly lower than the ANP level of the active INS (during edema) group (*p*=0.009) but did not show statistically significant differences when compared to ANP levels of either active INS group after resolution of edema or INS in remission group (*p*= 0.42 and 0.56, respectively). The genotypes and alleles frequencies of the 3 studied polymorphisms in active versus remission group did not show any significant differences (*p*>0.05 for all). However, among 100 INS patients, 18 had hypertension (7 maintained on captopril, 6 on losartan, and 5 combined captopril and nifedipine) the genotypes and alleles distribution in hypertensive compared to non-hypertensive patients did not show significant difference except for A2A2 genotype of ScaI polymorphism which was more frequent in hypertensive patients [*p*= 0.003], and A1 allele which was less frequent in hypertensive compared to non-hypertensive patients [*p*=0.012) (Table 3). Also, serum ANP levels did not show any significant differences between genotypes and alleles of the three studies' polymorphisms except for the A1A1 genotype of ScaI polymorphism which had higher ANP levels compared to the other 2 genotypes Table 4. ANP level distribution according to degree of edema did not show significant difference between patients with 1-puffy eyes+ LL edema + ascites (ANP 132.6±61.3 ng/ml), 2-patients with edema As 1 + scrotal or vulval edema (ANP level 127.01±49.2 ng/ml), 3-patients with edema as 1 or 2 +pleural effusion (ANP 125.4±41.8 ng/ml). Also; urinary sodium levels in the 3 groups of edema degree showed no significant difference. Higher serum ANP on admission was associated with a longer duration of edema resolution (*r*=0.79, *p* < 0.001). Urinary sodium levels and GFR in edematous patients were significantly lower while ANP levels were significantly higher than same patients after edema resolution (*p*< 0.001, 0.002, and < 0.001, respectively).

**Table 2:** Clinical, laboratory, and radiological data of the 2 groups of INS children

	Active NS (***N***=60) mean± SD	INS in remission (***N***=40) mean± SD	***P***
**Age at sampling (years)**	6.4±2.8	6.7±2.5	0.7
**Sex: male/female**	46/14	36/4	0.09
**Age at diagnosis (years)**	3.6±1.8	4.7±1.9	**0.004**
**Duration of illness (months)**	34.9±27.9	23.8±23.3	**0.04**
**Family history: yes/no**	5/55	2/38	0.7
**Days to resolution of edema:**	12.08±4.5	---------	-------
**Serum creatinine (mg/dl)**	0.55±0.2	0.45±0.2	**0.008**
**GFR (ml/min/1.73**^**2**^**)**	116.5±52.8	164.7±48.5	**<0.001**
	143.7±38.8		
**Serum albumin (g/dl)**	1.8±0.3	3.5±0.9	**<0.001**
**Serum cholesterol (mg/dl)**	418.2±103.9	353.7±128.9	**0.007**
**U pr/cr ratio**	4.4±1.6	0.18±0.15	**<0.001**
**Urinary sodium (mMol/L):**	During edema: 20.9±8.6	36.5±10.5	**<0.001**
	Resolved edema: 42.6±16.1		**0.04**
**Hypertension (diagnosed before enrollment in study)**	13 (21.7%)	5 (12.5%)	0.24
**Degree of edema:**			
**1-Puffy eyes+ LL edema + ascites**	25 (41.7%)	--------------	
**2-As 1 + scrotal or vulval edema**	30 (50%)		
**3-1 or 2 + pleural effusion**	5 (8.3%)		
**Types of NS:**			**<0.001**
Initial attack only:	32 (53.3%)	10 (25%)	
FRNS	4 (6.7%)	2 (5%)	
SDNS	4 (6.7%)	13 (32.5%)	
IFRNS	0	8 (20%)	
SRNS	20 (33.3%)	7 (17.5%)	
**Renal pathology:**			0.07
Not done	32 (53.3%)	30 (75%)	
MCD	9 (15%)	7 (17.5%)	
FPGN	5 (8.3%)	1 (2.5%)	
MN	4 (6.7%)	0	
FSGS	10 (16.7%)	2 (5%)	
**Plasma ANP** (pg/ml)**:**	During edema: 129.2±53.4	91.6±33.5	**<0.001**
	Resolved edema:102.3±29.75		0.097

*INS* idiopathic nephrotic syndrome, *SD* standard deviation, *GFR* glomerular filtration rate, *U pr/cr* urinary protein to creatinine ration, *FRNS* frequent relapsing nephrotic syndrome, *SDNS* steroid-dependent nephrotic syndrome, *IFRNS* infrequent relapsing nephrotic syndrome, *SRNS* steroid-resistant nephrotic syndrome, *MCD* minimal change disease, *FPGN* focal proliferative glomerulonephritis, *MN* membranous nephropathy, *FSGS* focal segmental glomerulosclerosis, *ANP* atrial natriuretic peptide, *IQR* interquartile range

Bold values are the statistically significant values

**Table 3:** Genotypes and alleles in the hypertensive compared to non-hypertensive patients

	Hypertensives (***N***=18) ***N*** (%)	Non-hypertensives (***N***=82) ***N*** (%)	***P***, OR (95% CI)
**ANP (A2843G)**			0.35
**Genotypes:**			
**AA**	12 (66.7%)	53 (64.6%)	
**AG**	6 (33.3%)	21 (25.6%)	
**GG**	0	8 (9.8%)	
**Alleles:**			
**A allele**	30 (83.3%)	127 (77.4%)	0.44
**G allele**	6 (16.7%)	37 (22.6%)	
**NPRC C (-55)**			0.43
**Genotypes:**			
**AA**	0	7 (8.5%)	
**AC**	5 (27.8%)	21 (25.6%)	
**CC**	13 (72.2%)	54 (65.9%)	
**Alleles:**			
**A allele**	5 (13.9%)	35 (21.3%)	0.3
**C allele**	31 (86.1%)	129 (78.7%)	
**ScaI gene polymorphism**			**0.008**
**Genotypes:**			
**A1A1**	1 (5.6%)	5 (6.1%)	0.9
**A1A2**	0	30 (36.6%)	0.03
**A2A2**	17 (94.4%)	47 (57.3%)	**0.003,12.6 (1.6-99.7)**
**Alleles:**			
**A1 allele**	2 (5.6%)	40 (24.4%)	**0.012, 0.18 (0.04-0.79)**
**A2 allele**	34 (94.4%)	124 (75.6%)	

*ANP* atrial natriuretic peptide, *NPRC* natriuretic peptide receptor clearance, *OR* odds ratio, *CI* confidence interval

Bold values are the statistically significant values

**Table 4:** Serum ANP levels distribution in genotypes and alleles of the 3 studied single nucleotide polymorphism

	ANP (A2843G) genotypes			***P***
	AA	AG	GG	
**ANP level**	123.3±53.9	141.3±44.3	134.3±74.3	0.27*, 0.29**, 0.8 ***
	**ANP (A2843G) alleles**			
	**A**	**G**		
**ANP level**	126.5±52.2	138.3±56.1		0.3
	**NPRC C (-55) genotypes**			
	**AA**	**AC**	**CC**	
**ANP level**	137.3±24.7	138.3±71.6	126.02±50.1	0.8^, 0.5^^, 0.45^^^
	**NPRC C (-55) alleles**			
	**A**	**C**		
**ANP level**	137.9±56.3	127.5±52.6		0.4
	**ScaI gene polymorphism genotypes**			
	**A1A1**	**A1A2**	**A2A2**	
**ANP level**	192.5±43.3	112.6±37.9	130.5±56.2	***0.01***^***#***^**,** 0.1^##^, 0.8^**###**^
	**ScaI gene polymorphism alleles**			
	**A1**	**A2**		
**ANP**	137.2±53.4	127.02±53.1		0.4

*AA versus AG+GG, ** AG versus AA+GG, *** GG versus AA+AG. ^: AA versus AC+CC, ^^: AC versus AA+CC, ^^^ CC versus AA+AC. # A1A1 versus A1A2+A2A2, ## A1A2 versus A1A1+A2A2, ### A2A2 versus A1A1+A1A2. *ANP* atrial natriuretic peptide, *NPRC* natriuretic peptide receptor clearance, *SNPs* single nucleotide polymorphisms

Bold values are the statistically significant values

## Discussion

In this study, we investigated the role of plasma ANP concentration in edema pathogenesis in INS children. In addition, we explored for the first time the possible role of three polymorphisms of ANP genes in pathogenesis of edema in INS. We found that plasma ANP concentration was significantly higher in active NS group (during edema) than in NS in remission and the control group. This indicates that ANP plays a role in edema formation in active NS and there is some sort of renal resistance to ANP diuretic and natriuretic actions supported by the non-significantly elevated urinary sodium levels despite high ANP levels. In NS, resistance to serum ANP is an observation in experimental animals and humans [17, 18], these studies showed that ANP levels were frequently elevated in INS children than in healthy or remission children. Restoration of renal receptiveness to ANP in animal models of NS by albumin infusion indicates that renal hypo-responsiveness to ANP is a reversible functional condition rather than permanent cellular damage [19]. Moreover, Cataliotti et al. observed increased plasma ANP concentration that frequently correlated with the edema severity [20]. Increased plasma ANP concentration may be a compensatory mechanism to induce diuresis and natriuresis. The ANP-dependent natriuresis is blunted, reflecting a dampening of the ANP-dependent signaling mechanism, and resulting in failure of ANP to correct extracellular volume burden in nephrotic patients [21]. Jovanovitsh and colleagues [22] reported similar findings in 1995 and concluded that in the nephrotic syndrome group ANP was higher than in the control group. Creatinine clearance (Ccr), diuresis, and natriuresis were significantly lower during relapse than in remission. The increase in Ccr, diuresis, and natriuresis was observed during the infusion of albumin. ANP resistance is also a hallmark of edematous disease states such as congestive heart failure [23] and liver cirrhosis [24]. There are multiple limitations of the use of urinary indices in edematous patients including use of diuretics or angiotensin-converting enzyme inhibitors or angiotensin receptor blockers and dietary salt intake [25].

In 2008, a third theory explaining edema formation in INS other than both overfill and underfill theories suggested that massive proteinuria during relapse causes tubulointerstitial inflammation and release of local vasoconstrictors leading to a reduction in single-nephron glomerular filtration rate (GFR) and salt and water retention [10]. Also, some researchers reject the hypothesis of resistance of INS patients to ANP natriuretic action. In 2011, Gurgoze et al. reported no difference between 3 groups: children with SSNS, remission, and control groups, regarding plasma ANP levels and stated no impact of ANP on the pathogenesis of edema [26]. In addition; Dönmez et al. studied children with MCD during and after the resolution of edema, ANP values were not significantly different although they were found to be insignificantly high on admission. They suggested that INS patients may have experienced temporary hypovolemia that gave rise to water and salt retention, and on clinical stabilization, hypervolemia and hypernatremia never occurred [27].

For more exploration of the role of ANP in the pathogenesis of diseases with edema and hypertension, some of the polymorphisms of genes regulating ANP or its receptors' expression have been explored. A2843G promoter polymorphism of the ANP gene was reported to be associated with left ventricular hypertrophy in patients with hypertension [12] while The ScaI polymorphism of the ANP gene was reported to be an important additive genetic factor influencing neurohormonal activation and disease progression in severe heart failure (HF). The NPRC polymorphism is not an independent determinant of natriuretic peptide (NP) concentration in HF [11] while other ANP gene polymorphisms as the concurrence of the rs5068 and rs198389 minor alleles appear to confer a similar protective cardio-metabolic phenotype supporting the possibility of development of ANP-based peptide therapeutics for cardio-metabolic disease states [28]. However, in the current work, the three studied polymorphisms did not show a significant difference between degrees of edema or the different alleles and genotypes except for the A1A1 genotype of ScaI polymorphism which had higher ANP levels compared to the other 2 genotypes. The A2A2 genotype of ScaI polymorphism was also more frequent and the A1 allele was less frequent in hypertensive versus non-hypertensive patients. Nannipieri et al; indicated that ScaI mutated allele (A1) was significantly lower in hypertensive than in control subjects and in patients with macroalbuminuria compared with normoalbuminuric subjects [29]. However, Zorc-Plesković and colleagues [30] found a trend (*p*=0.07) towards an association between the A1A1 genotype of the ScaI gene polymorphism and childhood essential hypertension but statistical significance was not reached. The A1 allele has been reported to cause loss of the regular stop codon, leading to an extension of the human natriuretic peptide by two additional arginine residues [31], this circulating form of ANP might have a different biological activity, playing a selective role in the regulation of glomerular filtration rate and the genesis of hyperfiltration [32]. In 2015; Ogawa et al. reported that age-related elevation of plasma ANP levels preceded the development of chronic kidney disease (CKD) in the general population of Japan, raising a possibility for ANP being involved in the development of CKD [33].

To the best of our knowledge, these three polymorphisms have not been studied before in children with INS. However, our results are preliminary and need future studies on a larger number of patients and different ethnic groups.

Limitations of the current work lie in the fact that it is a single center study, small number of controls, did not assess plasma renin activity and aldosterone levels moreover, the need for larger patients’ numbers before universally approving these preliminary findings.

## Conclusion

Atrial natriuretic peptide is elevated in edematous children with INS but with blunted renal response Polymorphisms of genes regulating ANP levels and receptors do not seem to be implicated in edema formation except for the A1A1 genotype of ScaI polymorphism. These results are preliminary and further studies with a larger number of subjects are required to confirm these findings.

## Abbreviations

INS: Idiopathic nephrotic syndrome
ANP: Atrial natriuretic peptide
SNP: Single nucleotide polymorphism
UPC: Urinary protein creatinine
NPRC: Natriuretic peptide clearance receptor
Ccr: Creatinine clearance
SRNS: Steroid-resistant nephrotic syndrome
SDNS: Steroid-dependent nephrotic syndrome
FRNS: Frequent relapsing nephrotic syndrome
IFRNS: Infrequent relapsing nephrotic syndrome
SSNS: Steroid-sensitive nephrotic syndrome
LL: Lower limb
SBP: Systolic blood pressure
DBP: Diastolic blood pressure
ELISA: Enzyme-linked immunosorbent assay
PCR: Polymerase chain reaction
RFLP: Restriction fragment length polymorphism
DNA: Deoxyribonucleic acid
SPSS: Statistical Package for Social Sciences
GFR: Glomerular filtration rate
HF: Heart failure
